# Research Progress in Medical Biomaterials for Bone Infections

**DOI:** 10.3390/jfb16050189

**Published:** 2025-05-21

**Authors:** Tianxu Lian, Yiwei Wang, Pengfei Zheng

**Affiliations:** Department of Orthopaedics Surgery, Children’s Hospital of Nanjing Medical University, Nanjing 210029, China; 2021121753@stu.njmu.edu.cn (T.L.);

**Keywords:** bone infection, antibacterial biomaterials, metal ions, nanomaterials, drug-loaded systems

## Abstract

Bone infection is a debilitating condition characterized by inflammation of the bone and its marrow. It poses significant challenges in clinical practice due to its recalcitrant nature and difficulty in eradicating the infecting microorganisms. Recent advancements in the field of medical biomaterials have shown hope in the treatment of bone infections. This article reviews the research progress of medical biomaterials for anti-osteomyelitis in recent years, focusing on the mechanism of action, unique advantages, and application backgrounds of various materials. At the same time, we pay attention to the need for materials used in the treatment of osteomyelitis to promote bone healing.

## 1. Background

Bone infection, a common complication of fractures, surgical operations, and infections related to implants, is an acute or chronic inflammatory process involving bones and their structures. The mortality rate of bone infections has significantly decreased following the widespread use of antibiotics, yet new challenges have emerged due to increasing bacterial resistance [1]. The complexity of bone infections lies in their diverse and unpredictable symptoms, and their treatment faces numerous difficulties. Firstly, the variety of pathogenic bacteria, with different species exhibiting varying sensitivity to antibiotics, poses a challenge in selecting the appropriate antibiotic. Moreover, the method of antibiotic use and related issues can also affect the treatment outcomes for patients with bone infections [2]. Secondly, bone infections are often accompanied by bone destruction and necrosis, necessitating complex surgical interventions, and the success rate of these surgeries is influenced by multiple factors such as the patient’s physical condition, the extent of the infection, and surgical technique. Furthermore, even after treatment, bone infections can recur, causing long-term pain and distress to patients [3]. In response to the treatment difficulties of bone infections, researchers are continuously exploring new methods and technologies. Among these, research on antimicrobial biomaterials has emerged as a significant direction. These biomaterials possess excellent biocompatibility, capable of inhibiting or killing pathogenic bacteria while promoting the regeneration and repair of bone tissue [4].

Medical biomaterials play a crucial role in the management of bone infections. These materials can be designed to possess specific properties that enhance their antimicrobial activity, osteoconductivity, and osteoinductivity. By incorporating antimicrobial agents, biomaterials can effectively kill or inhibit the growth of infecting microorganisms. This is also the main purpose of using medical biomaterials for bone infection, which can easily cause repeated infection and serious surgical trauma to patients after surgery. To reduce the morbidity of implant-related bacterial infections, biological medicine and the use of antibacterial materials are being given more attention [5]. Due to the increasing emergence of issues such as bacterial resistance, research on antibacterial materials is no longer limited to conventional anti-infection materials [6,7]. Apart from antibacterial properties, since the pro-inflammatory cytokines produced during bone infection often enhance the bone resorption capacity of mature osteoclasts and promote the differentiation of uninfected osteoclasts, leading to bone loss as a common complication of bone infection, the osteogenic properties of implant materials are also a major focus in the research direction of medical biomaterials for bone infections [8,9].

Addressing the aforementioned issues in bone infections, we have reviewed the recent research progress in medical biomaterials within this field, as well as the mechanisms by which these biomaterials exert antibacterial and osteogenic effects during the treatment of bone infections. This review provides a comprehensive summary and reflection on these contents, aiming to encourage further research in this area.

## 2. Methods

In the process of writing this review, we extensively consulted relevant domestic and foreign literature, including academic journal articles, theses, and monographs, to conduct a comprehensive analysis and summary of recent research progress on the biomedical materials used for bone infections. We systematically reviewed and discussed existing research achievements, theoretical foundations, and development directions in the field.

At the initial stage of the research, we conducted a preliminary search using databases such as PubMed and Web of Science with keywords like “bone infection” and “materials,” focusing on literature published in the last five years and prioritizing review articles; specifically, in the PubMed database, we performed a search using the query (bone infection) OR (osteomyelitis)) AND (materials), which yielded 12,150 results, and after further filtering for review articles published within the last five years, we obtained 647 search results. This helped us understand the current hotspots in material development and research, allowing us to establish the general framework of the article and identify the key aspects to be elaborated in each section. For instance, we noted the antibacterial and osteogenic properties of metal-based materials such as silver ions and magnesium ions.

After determining the structure of the article, we conducted targeted searches for each section, primarily using the PubMed database, with a focus on research articles, particularly foundational medical studies investigating material efficacy and mechanisms. We refined our search by incorporating performance-related keywords, such as (silver ions) OR (silver) AND (antibacterial) OR (anti-infection) AND (bone infection) OR (osteomyelitis), still limiting the timeframe to the past five years. We obtained 29,466 results, which were narrowed down to 4370 review articles and 638 clinical trial articles after screening. This search strategy enabled us to locate relevant literature for in-depth analysis and review writing. Additionally, we conducted similar searches in the CNKI (China National Knowledge Infrastructure) database to ensure a balanced inclusion of research progress from different regions, with diverse characteristics and focuses.

During the research, we observed that some materials had garnered attention for their properties well before the past five years. Therefore, in writing each section, we expanded the search scope to include literature from the past ten or even fifteen years. Additionally, we examined the references cited in the retrieved articles to ensure a more comprehensive understanding of the materials, thereby deepening the content of the review.

## 3. Antibacterial Biomaterials

### 3.1. Metal Ions

Significant progress has been made in the research of antibacterial metals and alloys in the field of implant materials in recent years, especially in the prevention and treatment of implant-associated infections (IAI). These materials can effectively reduce the risk of bacterial infection, improve the success rate of implants, and enhance the quality of life of patients through different antibacterial mechanisms. Metal and alloy materials can either be applied as coatings on the surface of implant materials to exhibit antibacterial effects, or certain metallic materials can be directly used as implants in the treatment of bone infections [10]. The antibacterial mechanisms of metallic ions that exhibit antibacterial properties share similarities yet also possess unique characteristics. For instance, titanium metal, which is commonly used in implant materials, does not inherently possess antibacterial properties. However, by incorporating antibacterial elements such as copper and silver, titanium can be endowed with antibacterial capabilities, making it more suitable for the treatment of bone infections. Surface modification of titanium metal with copper or silver, or the creation of titanium alloys containing silver or copper, can release copper ions and silver ions to achieve antibacterial effects [11]. Additionally, metal-based materials can achieve enhanced antibacterial properties through methods such as nano-modification [12]. Antibacterial metals and alloys hold immense potential as implant materials in preventing and treating IAI. Through intensive research into their antibacterial mechanisms, material properties can be further optimized to enhance their antibacterial efficacy.

#### 3.1.1. Silver Ions

Silver ions (Ag⁺) and their nanoparticles (AgNPs) have garnered significant attention and widespread application in various fields such as medicine, public health, and industry due to their unique antimicrobial properties. In a 2023 study, bone cement impregnated with silver nanoparticles (AgNPs) was used to test its antibacterial effects in a rat model of MRSA-induced osteomyelitis (“MRSA” stands for methicillin-resistant *Staphylococcus aureus*, a common antibiotic-resistant bacterium). The diameter of the MRSA growth inhibition zone on the AgNP-treated discs ranged from 4.1 to 13.3 mm, demonstrating antibacterial activity [13].

Silver ions interact with the phospholipids and thiol groups (-SH) in bacterial cell membranes, causing structural and functional damage. This disruption impairs the integrity and permeability of the cell membrane, leading to the leakage of intracellular contents and the influx of external harmful substances, ultimately resulting in bacterial death [14].

Upon entering bacterial cells, silver ions interact with proteins and DNA, disrupting their structures and functions. They interfere with the bacterial protein synthesis system, inhibiting the activity of crucial enzymes and thereby suppressing bacterial growth and reproduction. Additionally, silver ions can induce replication errors and damage to DNA, further exacerbating bacterial demise [14].

Silver ions react with certain molecules within bacterial cells, such as glutathione, generating substantial amounts of reactive oxygen species (e.g., superoxide anions, hydrogen peroxide). These ROS trigger oxidative stress within bacterial cells, damaging biomolecules like proteins and DNA, ultimately leading to bacterial death [15].

However, as mentioned earlier, with the advancement of nanotechnology, silver nanoparticles (AgNPs) have become a research hotspot in recent years due to their diversified antibacterial mechanisms [15]. Silver nanoparticles (AgNPs), owing to their minute size and high surface-to-volume ratio, can penetrate bacterial cell walls and membranes, directly acting on bacterial interiors [5]. Furthermore, AgNPs inhibit the formation of bacterial biofilms, preventing the establishment of difficult-to-eradicate bacterial colonies at infection sites [16]. This is particularly crucial in treating chronic infections and implant-associated infections [17].

**Figure 1:**
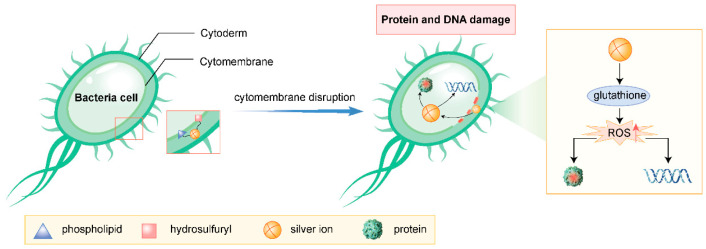
Antibacterial mechanism of silver ions (drawn by Adobe Illustrator).

Silver ions and their nanoparticles have extensive applications in the medical field. For instance, silver ion-infused antimicrobial dressings are used to treat sensitive wounds (e.g., burns, diabetic foot ulcers), promoting wound healing through their antimicrobial properties [18]. Additionally, silver ions are employed in the production of antimicrobial medical devices (e.g., catheters, heart valves), minimizing the risk of infection [19] (Figure 1).

#### 3.1.2. Strontium Ions

Strontium (Sr) is one of the essential trace elements in the human body, playing a role in promoting bone formation and inhibiting bone resorption during bone regeneration. By doping the surface of biomaterials or forming specific compounds, strontium ions can endow materials with antibacterial properties. Studies have shown that strontium ions can inhibit the growth and reproduction of bacteria through various mechanisms [20].

Firstly, strontium ions can disrupt the bacterial cell membrane structure, increasing its permeability, which leads to the leakage of internal bacterial substances and ultimately bacterial death. This mechanism enables strontium ions to exert significant inhibitory effects on a wide range of bacteria, including both Gram-positive and Gram-negative bacteria [21].

Secondly, strontium ions can also inhibit the formation of bacterial biofilms. Biofilms are protective structures formed by bacteria in their environment, enhancing bacterial drug resistance and survival ability. Strontium ions disrupt the structure and stability of biofilms by inhibiting the secretion of biofilm-forming substances such as polysaccharide intercellular adhesin (PIA) by bacteria, thereby reducing bacterial survival rates [22].

Furthermore, strontium ions can activate the immune response of host cells, promoting the polarization and activation of macrophages and enhancing the body’s ability to clear bacteria. Studies have demonstrated that strontium ions can activate the Akt and Erk signaling pathways, promoting the polarization of macrophages towards the M2a phenotype and the secretion of growth factors such as PDGF-BB, which facilitates vascularization and bone integration processes. The Akt and Erk pathways are central signaling cascades regulating cell survival, proliferation, and metabolism, with Akt primarily mediating growth factor and insulin responses, while Erk drives mitogen-activated proliferation, both implicated in cancer and metabolic diseases. These effects not only help to inhibit bacterial infections but also promote the regeneration and repair of bone tissue [23].

#### 3.1.3. Cobalt Ions

Cobalt ions, as a promising antimicrobial material, have demonstrated their unique application value in the field of bone infection treatment. Their antimicrobial mechanism, primarily based on the destruction of bacterial cell structure and the inhibition of bacterial metabolic activities, has been supported by multiple studies and elaborated in relevant literature [24].

Cobalt ions can penetrate into the interior of bacterial cells and interact with internal biomolecules such as DNA, RNA, and enzymes. This interaction disrupts the genetic material of bacteria, interfering with their normal metabolic processes and leading to bacterial death. Additionally, cobalt ions can affect the permeability of bacterial cell membranes, compromising the barrier function of these membranes. This damaged membrane results in the loss of nutrients from within the bacteria and the ingress of external harmful substances, further exacerbating bacterial death [25].

In the treatment of bone infections, the application of cobalt ions is not limited to being a direct antimicrobial agent. Researchers have also formed composites with antimicrobial properties by combining cobalt ions with other materials. These composites can be utilized in medical devices such as fracture fixation implants and bone defect repair materials. By continuously releasing cobalt ions, these composites effectively inhibit the growth and reproduction of bacteria, thereby reducing the risk of bone infections [26].

For example, a study constructed a novel multifunctional implant (Ag@Co/β-TCP) composed of cobalt, silver nanoparticles, and bioceramic β-tricalcium phosphate (β-TCP) through press molding and polydopamine-assisted modification. This implant exhibited excellent antimicrobial performance against both Gram-positive (*Staphylococcus aureus*) and Gram-negative (*Escherichia coli*) pathogens. Furthermore, in vitro cellular experiments demonstrated that Ag@Co/β-TCP possessed good biocompatibility and cell growth-promoting abilities. These advantages enable the functionalized bioceramic Ag@Co/β-TCP to achieve both antimicrobial and osteogenic purposes without the use of antibiotics, providing new insights into solving infection issues arising during implant surgeries [27].

Cobalt ions have demonstrated unique advantages and potential as antimicrobial materials in the treatment of bone infections. Their antimicrobial mechanism is clear and well-defined, with strong, broad-spectrum antimicrobial activity. By forming composites with antimicrobial properties through combinations with other materials, cobalt ions offer new ideas and methods for the treatment of bone infections. However, the application of cobalt ions also requires attention to their safety and effectiveness to avoid potential side effects from excessive or prolonged use.

Silver ions (Ag⁺), strontium ions (Sr^2^⁺), and cobalt ions (Co^2^⁺), as representative antibacterial metal ions, each exhibit distinct mechanisms yet share common characteristics of broad-spectrum antimicrobial activity through multiple pathways, including bacterial membrane disruption, metabolic interference, and biofilm inhibition. These three ions not only demonstrate the core value of metal ions in bone infection treatment—achieving a balance between sustained antibacterial effects and biocompatibility through controlled ion release—but also establish important paradigms for developing novel antimicrobial materials. First, the multi-target antibacterial mechanisms of metal ions can effectively circumvent bacterial resistance; second, their composite strategies with biomaterials (e.g., nanoparticle modification, ceramic doping) provide clear directions for designing implants with integrated antibacterial and osteogenic functions; most importantly, their interactions with host immune systems (e.g., strontium-mediated macrophage polarization) suggest that future material development should simultaneously target direct bactericidal activity and immunomodulation. These findings offer critical insights for research on other functional metal ions (e.g., copper, zinc)—ideal antibacterial ion systems should integrate triple properties of efficient bactericidal capacity, osteogenic activity, and host response regulation, employing material science approaches to achieve spatiotemporally controlled release for synergistic infection control and tissue regeneration.

### 3.2. Nanomaterials

Nanomaterials can achieve antibacterial effects through multiple mechanisms, including but not limited to disrupting bacterial cell membranes, inhibiting bacterial growth and dissemination, inducing oxidative stress responses in cells, and utilizing photothermal effects [28].

Firstly, the sharp edges and corners of nanomaterials act like “nanoknives,” readily puncturing bacterial cell membranes upon contact, causing the leakage of cellular contents and subsequent bacterial death. Furthermore, nanomaterials can release metallic ions (such as silver and copper ions) or reactive oxygen species (like superoxide anions and hydroxyl radicals), damaging critical components within bacterial cells like lipids, proteins, and DNA, thereby inhibiting bacterial growth and reproduction [29].

Secondly, the photothermal effect of nanomaterials offers a novel approach for antibacterial applications. For instance, gold nanoparticles and molybdenum disulfide nanosheets can absorb near-infrared light and convert it into heat energy, effectively eradicating bacteria and even tumor cells [30]. Similarly, nanosized titanium dioxide and zinc oxide, upon excitation by ultraviolet light, generate highly oxidative reactive oxygen species that can oxidize and kill bacteria [31].

**Figure 2:**
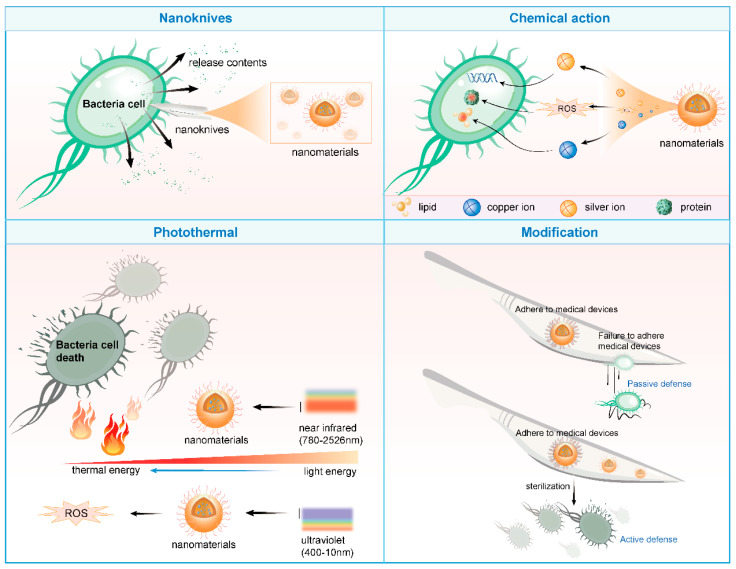
Antibacterial mechanism of nanomaterials (drawn by Adobe Illustrator).

Moreover, nanostructured materials can leverage biomimetic principles to pattern medical device surfaces at the nanoscale, preventing bacterial adhesion and achieving passive defense [32]. Simultaneously, coating medical device surfaces with nanomaterials that possess bactericidal properties enables active bacterial killing. This dual-defense mechanism significantly enhances the anti-infective safety of medical devices [33,34] (Figure 2).

#### 3.2.1. Nano-Zinc Oxide

The direct interaction between nano-zinc oxide (ZnO NPs) and bacterial cell membranes is one of the primary mechanisms of its antibacterial effect. Research has shown that ZnO NPs can significantly affect the cytoplasmic membrane of bacteria within an extremely short period (e.g., 15 min), causing membrane damage in over 70% of the cells. ZnO NPs adsorb onto the negatively charged bacterial surface through electrostatic interactions, where their positive charges bind with the negative charges on the cell surface, forming a charge gradient that leads to membrane rupture. Additionally, the dissolution of ZnO in solution releases Zn^2^⁺ ions, which enter bacterial cells through passive diffusion, further disrupting the membrane structure [35].

Under light or biological conditions, nano-zinc oxide can generate electron–hole pairs, triggering a series of redox reactions that produce reactive oxygen species (ROS) such as hydroxyl radicals (OH·) and superoxide anions (O_2_). These ROS induce oxidative stress in bacterial cells, inhibiting protein synthesis and DNA replication, ultimately leading to bacterial death [36,37]. Furthermore, Zn^2^⁺ ions can oxidize sulfur groups within bacterial cells, inhibiting key enzymes like glycolytic enzymes and exacerbating cellular damage. Moreover, recent studies have demonstrated that, in addition to traditional chemical synthesis, plant-mediated green synthesis can also produce nano-zinc oxide materials with better photocatalytic and antibacterial properties [38].

Nano-zinc oxide not only directly damages cell membranes but also interacts with bacterial proteins and DNA to further inhibit bacterial growth and reproduction. ZnO NPs adsorb onto bacterial surfaces, interacting with the peptidoglycan layer in the cell wall, causing cell wall lysis. Concurrently, Zn^2^⁺ ions enter bacterial cells and bind to DNA, disrupting its double-helical structure and preventing DNA replication and transcription [39]. In a 2020 study, zinc oxide nanoparticles (ZnO NPs) were used as a surface coating for NiTi wires, demonstrating strong antibacterial effects. The material reduced the count of S. mutans (*Streptococcus mutans*) to zero within 2 h. It also exhibited significant inhibitory effects against Gram-positive *S. aureus* and *P. aeruginosa* and Gram-negative *E. coli* and Pseudomonas. The study revealed that ZnO NPs interact with bacterial proteins and DNA, efficiently suppressing bacterial growth and reproduction. Nano-zinc oxide can also indirectly exert an antibacterial effect by activating the host immune system. Studies have demonstrated that ZnO NPs enhance the antibacterial activity of polymorphonuclear neutrophils (PMNs), including promoting PMN migration, phagocytic efficiency, and ROS production [40]. ZnO NPs release Zn^2^⁺ ions, stimulating PMN chemotaxis and the release of pro-inflammatory cytokines (e.g., TNF-α, IL-1β, IL-6), further augmenting the antibacterial capabilities of immune cells.

The antibacterial mechanism of nano-zinc oxide is not a single pathway but a result of the synergistic action of multiple mechanisms [41]. Its antibacterial effect is influenced by various factors, including the concentration, size, morphology, surface charge, and duration of exposure of the nanoparticles. Additionally, the antibacterial mechanism of nano-zinc oxide may be influenced by external conditions such as bacterial species and growth environments.

#### 3.2.2. Nano-Titanium Dioxide

In exploring the antibacterial mechanism of titanium dioxide nanomaterials (TiO_2_ NPs) applied in bone infections, we conducted an in-depth analysis of their unique antibacterial properties and potential modes of action. The experimental results demonstrate that TiO_2_ NPs exhibit potent antibacterial activity against a variety of bacteria, including common pathogens that cause bone infections, such as *Staphylococcus aureus* (*S. aureus*) and *Escherichia coli* (*E. coli*) [42]. This antibacterial effect is closely related to the concentration of TiO_2_ NPs (titanium dioxide nanoparticles) and the antibiotics used in combination, with higher concentrations leading to enhanced antimicrobial performance. In a 2023 study on the antibacterial properties of green-synthesized ZnO and TiO_2_ NPs, researchers tested TiO_2_ NPs both alone and in combination with antibiotics at varying concentrations (10 mg/mL, 20 mg/mL, 30 mg/mL, and 40 mg/mL). They found that the highest ZOI (zone of inhibition) was achieved when TiO_2_ NPs were combined with antibiotics at 40 mg/mL. In contrast, chemically synthesized TiO_2_ NPs alone exhibited a ZOI in the range of 5–7 mm, demonstrating significant antibacterial activity [43].

The antibacterial mechanism of TiO_2_ NPs is primarily attributed to the oxidative stress they generate. Under light conditions, TiO_2_ NPs can absorb light energy and convert it into chemical energy, subsequently producing reactive oxygen species (ROS) with strong oxidizing properties. These ROS attack bacterial cell membranes, disrupting their integrity and causing the leakage of internal bacterial substances, ultimately leading to bacterial death [37,44]. Additionally, TiO_2_ NPs may further exacerbate the bactericidal effect by interfering with bacterial energy transport, inhibiting cell wall/membrane synthesis, and disrupting protein function through multiple pathways [45].

Notably, the antibacterial properties of TiO_2_ NPs offer significant advantages in the treatment of bone infections. Due to the unique physiological environment and complex anatomical structure of bone tissue, traditional antibiotics often fail to achieve effective treatment. However, TiO_2_ NPs, with their nanoscale size effect and excellent biocompatibility, can more easily penetrate into bone tissue, coming into direct contact with bacteria and exerting an antibacterial effect. Furthermore, the antibacterial mechanism of TiO_2_ NPs is less likely to induce bacterial resistance, providing a new strategy for the treatment of bone infections.

Both zinc oxide nanoparticles (ZnO NPs) and titanium dioxide nanoparticles (TiO_2_ NPs) demonstrate significant antibacterial properties, yet exhibit distinct mechanisms and characteristics. ZnO NPs primarily exert rapid antibacterial effects through multiple pathways, including direct bacterial membrane disruption, Zn^2^⁺ ion release, and ROS generation, maintaining activity even in light-free conditions, making them suitable for deep bone infection treatment. In contrast, TiO_2_ NPs’ antibacterial efficacy largely depends on photocatalytically generated ROS, showing remarkable effectiveness against drug-resistant bacteria without inducing resistance, though their application in light-deficient deep tissues remains limited. Regarding toxicity, ZnO NPs require careful control of Zn^2^⁺ release to avoid potential cytotoxicity, while TiO_2_ NPs may pose risks of nanoparticle accumulation upon prolonged retention. For future development of dual-functional biomaterials with both antibacterial and osteogenic properties, a strategic combination of their advantages or performance optimization through surface modification could better address the therapeutic demands of bone infections.

### 3.3. Antibacterial Peptides

#### 3.3.1. Antimicrobial Peptide LL-37 Human

LL-37 is an amphipathic helical peptide composed of 37 amino acid residues, initially discovered in human leukocytes and testes, and subsequently found in other cells, tissues, and bodily fluids. It is produced by the cleavage of the C-terminal end of its precursor protein, hCAP-18, and has a widespread distribution and expression, mainly present in immune cells, epithelial cells, skin, and oral mucosa [46]. LL-37 exists in different forms in different solutions, exhibiting a secondary disordered structure and forming oligomers in pure water, but adopting an α-helical structure in lipid models, micellar solutions, and ionic solutions. Studies have shown that the α-helical structure is more effective in killing bacteria [47].

LL-37 exhibits potent antibacterial activity against both Gram-positive and Gram-negative bacteria. The cell walls of Gram-negative bacteria contain large amounts of lipopolysaccharide (LPS), while the cell walls of Gram-positive bacteria are coated with lipoteichoic acid. Both are negatively charged, and LL-37, which is positively charged, readily binds to the negatively charged lipoteichoic acid or LPS on the bacterial cell wall. This binding leads to the destruction of the cell wall, outflow of intracellular substances, and disintegration of the cell wall, ultimately causing bacterial death [46].

The formation of biofilms is an important resistance mechanism of bacteria. Bacteria adhere to surfaces, secreting polysaccharides, fibrin, and other substances to encapsulate themselves and form large aggregates of membrane-like structures. LL-37 not only directly kills free-floating bacteria but also inhibits the formation of biofilms, thereby effectively clearing bacteria enclosed in biofilms and reducing the risk of reinfection [48].

In addition to its direct antibacterial activity, LL-37 can also indirectly resist infection by regulating immune responses. It activates innate mucosal immune responses, promotes the chemotaxis of immune cells, and releases cytokines, thereby enhancing the body’s natural immunity. Furthermore, LL-37 can balance tissue inflammation, inhibit excessive inflammatory responses, and promote wound healing [49].

#### 3.3.2. Cecropins

The antibacterial mechanism of cecropin antimicrobial peptides primarily involves the disruption of bacterial cell membranes. Specifically, cecropin antimicrobial peptides, through the formation of a special amphipathic helical structure, are able to target non-polar lipid cell membranes [50]. Upon binding to the membrane, they create ion-permeation channels, causing cell depolarization and leading to irreversible cell lysis and death [51].

The amphipathic helical structure of cecropin antimicrobial peptides enables them to specifically recognize and bind to the lipid bilayer of bacterial cell membranes. This binding action is achieved through the interaction between the hydrophobic side chains of cecropin antimicrobial peptides and the non-polar lipids on the cell membrane.

Once bound to the cell membrane, cecropin antimicrobial peptides insert into the membrane, forming ion-permeation channels. These channels allow a large influx of cations (such as Na⁺, K⁺, etc.) and water molecules into the cell, disrupting the intracellular ion balance and causing cell depolarization [50].

As the intracellular ion concentration rises and water molecules flood in, the osmotic pressure of the bacterial cell changes, leading to membrane rupture and cell lysis. Ultimately, the bacterial cell dies due to the loss of its integrity [51].

Furthermore, cecropin antimicrobial peptides can penetrate the lipopolysaccharide (LPS) barrier, affecting the permeability of the outer membrane (OM) and cytoplasmic membrane (CM) [52]. After membrane permeabilization, a large influx of cationic antimicrobial peptides enters the cell, damaging various bacterial mechanisms, such as inhibiting transcription and DNA replication, cytokinesis, cell wall biosynthesis, enzyme activity, and protein synthesis. These effects further enhance the antibacterial efficacy of cecropin antimicrobial peptides [53].

#### 3.3.3. Melittin

Bee venom peptide (Melittin) is a polypeptide composed of 26 amino acid residues, with a molecular weight of approximately 2840 and accounting for about 50% of the dry weight of bee venom. Its molecular structure is characterized by four amino acid residues carrying positive charges at the C-terminus and two amino acid residues carrying positive charges at the N-terminus, giving the entire molecule six positive charges. Additionally, the N-terminus of Melittin is mainly hydrophobic, while the C-terminus is hydrophilic and strongly alkaline [54].

As a cationic antimicrobial peptide, Melittin exerts its antibacterial effect mainly through non-specific binding to the bacterial cell membrane. This binding action lacks high selectivity, making it difficult for bacteria to develop resistance to Melittin through simple mutations or peptide modifications [55]. The antibacterial mechanism of Melittin primarily includes the following aspects: ① Disruption of the cell membrane: Melittin interacts with the negative charges on the bacterial cell membrane using its positive charges and then inserts into the membrane. Due to its hydrophobic and hydrophilic properties at both ends, Melittin can disrupt the phospholipid bilayer structure of the cell membrane, increasing membrane permeability and ultimately causing leakage of cellular contents, thereby achieving a bactericidal effect. ② Inhibition of cellular metabolism: By binding to the cell membrane, Melittin can also inhibit metabolic activities within bacterial cells, such as the synthesis of DNA, RNA, and proteins, further hindering bacterial growth and reproduction. ③ Induction of autolysis: Melittin can also induce autolysis in bacterial cells by activating the intracellular autolytic enzyme system, leading to self-digestion and death of the cells.

Melittin exhibits broad-spectrum antibacterial activity, inhibiting the growth and reproduction of various Gram-negative and Gram-positive bacteria, particularly showing significant antibacterial effects against penicillin-resistant Staphylococcus aureus [54]. Additionally, Melittin possesses various biological activities, such as antifungal, antiviral, and antitumor properties, making it have broad application prospects in the medical and healthcare fields [55].

### 3.4. Enzymes

#### 3.4.1. Lysozyme

Lysozyme, as a natural antimicrobial protein, exhibits unique advantages in the treatment of bone infections. It not only effectively lyses peptidoglycan in bacterial cell walls, demonstrating significant bactericidal effects against Gram-positive bacteria, but also enhances the inhibition of Gram-negative bacteria through synergistic interactions with other antimicrobial agents [56]. Recent advances in innovative carrier design and delivery systems have further expanded their application potential in infected bone defect repair [57]. For instance, a chitosan scaffold loaded with lysozyme and CCMV-YGFGG nanoparticles exemplifies a sophisticated sequential release strategy, where rapid release of lysozyme provides early-stage infection control, while sustained release of osteogenic peptides from viral vectors promotes bone regeneration, effectively addressing the temporal conflict between infection eradication and tissue repair [58].

Another delivery system based on alginate dialdehyde-gelatin (ADA-GEL) hydrogels incorporates lysozyme-loaded mesoporous cerium-doped silica-calcium nanoparticles (Lys-Ce-MSN), creating a multifunctional composite material. This system not only preserves lysozyme’s antimicrobial efficacy but also modulates the inflammatory microenvironment via cerium ions’ antioxidant activity, while a sustained release of calcium and silicon ions enhances apatite mineralization and bone matrix deposition [59].

Notably, these advanced systems exhibit additional benefits beyond antimicrobial action, such as promoting osteoblast proliferation/differentiation and potentially inhibiting osteosarcoma cell growth, offering new therapeutic avenues for complex bone infections with comorbid malignancies. However, challenges remain in optimizing lysozyme’s stability when in vivo and on a large-scale production. Current studies confirm its promising role in comprehensive infected bone defect therapy when combined with rational material design and delivery strategies.

#### 3.4.2. Glucose Oxidase (GOx)

Glucose oxidase (GOx), a biocatalytic enzyme, has emerged as a unique therapeutic agent for infected bone defects, primarily leveraging its ability to modulate local glucose metabolism and generate reactive oxygen species (ROS).

In pathological microenvironments like diabetic bone infections, GOx specifically catalyzes the oxidation of β-D-glucose to gluconic acid and hydrogen peroxide (H_2_O_2_), depleting a critical energy source for pathogens while creating an acidic milieu unfavorable for microbial survival. Crucially, as demonstrated in recent studies, GOx synergizes with transition metal ions (e.g., Cu^2+^) to further convert H_2_O_2_ into highly toxic hydroxyl radicals (•OH) via Fenton-like reactions, achieving photo-enhanced chemodynamic therapy with a remarkable turnover [60].

Additionally, GOx-mediated metabolic intervention regulates immune homeostasis by polarizing macrophages toward an anti-inflammatory phenotype (M2), complementing its direct antimicrobial effects. Its substrate-dependent activation in hyperglycemic diabetic environments ensures targeted therapy while minimizing systemic toxicity. Despite unresolved challenges in long-term stability and implant compatibility, GOx’s dual metabolic-chemodynamic mechanism offers a novel paradigm for treating complex bone infections, particularly in diabetic osteointegration disorders [61].

### 3.5. Polysaccharides

#### 3.5.1. Chitosan (CS)

Chitosan (CS), a natural cationic polysaccharide, demonstrates distinctive antimicrobial properties in infected bone defect therapy, with mechanisms closely tied to its material characteristics and microbial microenvironment [62]. The protonation of amino groups into NH^3+^ under acidic conditions (pH ≈ 6.0) enables electrostatic interactions with negatively charged bacterial components (e.g., lipopolysaccharides, teichoic acids), disrupting membrane integrity and causing cytoplasmic leakage [63]. CS also penetrates cells to bind DNA and chelate essential metal ions (e.g., Mg^2+^, Ca^2+^), further impairing microbial metabolism [64]. Its antimicrobial efficacy is molecular weight (MW)-dependent: high-MW CS (>300 kDa) forms physical barriers on Gram-positive bacteria (e.g., *Staphylococcus aureus*), while low-MW CS enters Gram-negative bacteria (e.g., *Escherichia coli*) to interfere with metabolism. This differential action ensures broad-spectrum coverage against polymicrobial infections. CS’s thermosensitive activity (enhanced at 4–37 °C) aligns with the febrile nature of infected sites, augmenting its clinical relevance [64]. Although solubility limitations (pH < 6.5) pose challenges, optimizing the deacetylation degree (DD) and MW can significantly improve efficacy, guiding the design of CS-based bone repair materials [65].

#### 3.5.2. Sodium Alginate (SA)

Sodium alginate (SA), an anionic polysaccharide composed of β-D-mannuronic acid (M) and α-L-guluronic acid (G) units, exhibits notable antimicrobial and osteogenic properties in bone infection management [66]. Its carboxyl groups interact electrostatically with bacterial membranes, while pH-responsive behavior (enhanced activity at pH 5.5–6.5) targets inflammatory microenvironments. SA’s ability to chelate metal ions (e.g., Ca^2+^, Zn^2+^) forms stable hydrogels for drug delivery and scaffold fabrication [67]. High-G-content SA shows superior activity against Gram-positive bacteria (e.g., *S. aureus*), likely due to enhanced metal-binding capacity, disrupting microbial metabolism. SA’s biocompatibility and gelation properties make it an ideal carrier for antibiotics (e.g., gentamicin) or nanoparticles (e.g., AgNPs), enabling synergistic effects [68,69]. In bone repair, SA promotes osteoblast mineralization via Ca^2+^ release and supports vascularization through its porous structure [70]. While standalone SA has limited antimicrobial potency, chemical modifications (e.g., oxidation, sulfation) or composites (e.g., with CS) can augment its dual functionality, offering innovative solutions for multifunctional bone grafts.

## 4. Antibacterial Materials with Osteogenic Properties

### 4.1. Metal-Based Materials

#### 4.1.1. Magnesium

The application of magnesium metals and their alloys in the medical field has a long history, particularly in orthopedics, where magnesium metals are considered ideal bone graft materials due to their unique properties [71]. However, the mechanism by which magnesium promotes bone formation is not yet fully understood. Nevertheless, magnesium’s application in bone grafts remains limited by its suboptimal mechanical strength and corrosion resistance [72]. Magnesium ions exhibit various biological effects in organisms, one of which is the promotion of new bone formation. Studies have found that during the degradation process of magnesium metal implants in organisms, the magnesium ion concentration around the implants significantly increases, a phenomenon that promotes the formation of new bone tissue. Additionally, the addition of magnesium ions to bone graft materials can also effectively promote new bone formation [73].

To further explore the mechanism by which magnesium promotes bone formation, researchers have conducted numerous in vitro experiments. The experiments revealed that magnesium ions at appropriate concentrations (such as 6–10 mM) can significantly enhance the viability and differentiation of osteoblasts, while excessively high concentrations of magnesium ions (such as 18 mM) inhibit the viability and differentiation of osteoblasts. This result indicates that the effect of magnesium ions on osteoblasts is concentration-dependent [74].

Recent studies have shown that the PI3K/Akt signaling pathway plays a crucial role in regulating the proliferation and differentiation of osteoblasts. The PI3K/Akt signaling pathway is a central pathway regulating cell growth, proliferation, metabolism, and survival. Compared to the previously mentioned Akt pathway, it places greater emphasis on the upstream activation mechanism, where PI3K acts as the initiating kinase, generating PIP3 to activate Akt. This pathway can be stimulated by growth factors, insulin, and other signals, ultimately promoting cell survival and inhibiting apoptosis through phosphorylation of downstream targets (e.g., mTOR, GSK-3β). Researchers hypothesize that the mechanism by which magnesium metals promote bone regeneration may be through the activation of the PI3K/Akt signaling pathway by their degradation products, magnesium ions. To verify this hypothesis, the researchers conducted a series of experiments. The experimental results showed that under the action of magnesium ions at appropriate concentrations, the expression level of phosphorylated Akt (p-Akt) significantly increased. However, when a specific inhibitor of the PI3K/Akt signaling pathway was added to the magnesium ion group, the activation effect of magnesium ions on the PI3K/Akt signaling pathway was significantly reversed, and the biological behaviors of osteoblasts, such as adhesion, viability, and differentiation, were also inhibited. This result further confirms the key role of the PI3K/Akt signaling pathway in magnesium-promoted bone formation [75].

**Figure 3:**
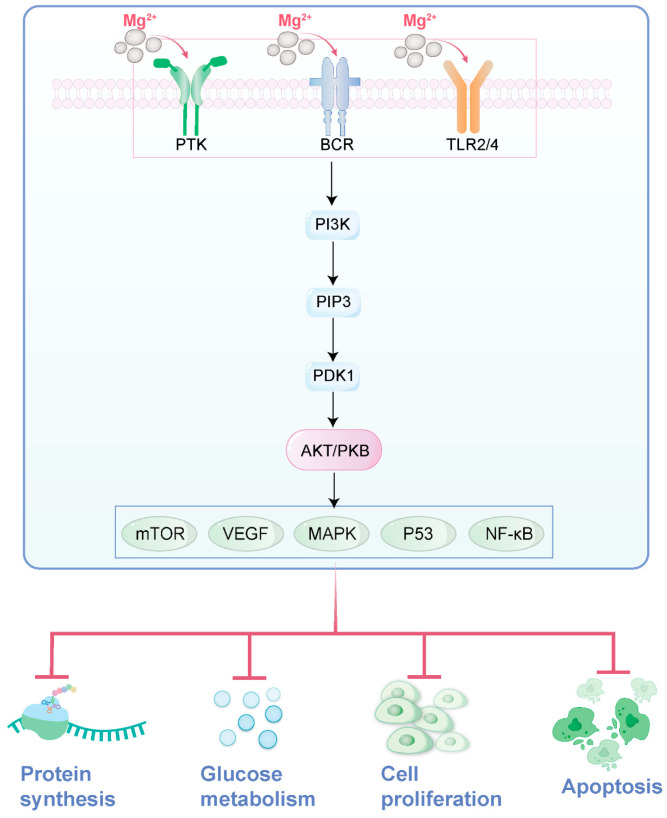
The role of magnesium ions in the PI3K/Akt signaling pathway (drawn by Adobe Illustrator).

Magnesium metals have also made significant progress in the application of fracture healing. Researchers found that after implanting magnesium metals into the femoral marrow cavity of rats, a large amount of new bone formed around the femur. This result not only further confirms the osteogenic effect of magnesium metals but also provides strong support for their clinical application in fracture repair (Figure 3).

#### 4.1.2. Zinc

Zinc is one of the indispensable elements for maintaining bone health. It not only participates in bone growth and development but also plays a crucial role in bone remodeling and the prevention of osteoporosis. As age increases, bone mineral density gradually declines, making it particularly important to maintain adequate zinc levels in the body to preserve bone health [76,77].

Firstly, zinc plays a significant role in the proliferation, differentiation, and apoptosis of osteoblasts. Adequate zinc can stimulate the proliferation of osteoblasts, promote the differentiation of stem cells into pre-osteoblasts, and subsequently form mature osteoblasts. This process is crucial for the formation of new bone. Zinc also indirectly promotes bone health by influencing the synthesis of insulin-like growth factor-1 (IGF-1), a protein molecule that plays an important role in maintaining bone health. Zinc stimulates the synthesis of IGF-1, thereby further promoting osteogenic activity [77].

Secondly, zinc enhances the activity of alkaline phosphatase (Alkaline phosphatase), an enzyme that stimulates osteoblasts to initiate new bone formation. The presence of zinc significantly increases its activity, thereby accelerating the process of new bone formation [78]. In a study of patients with traumatic fractures, those who took 50 mg of zinc daily showed positive effects on bone callus formation after 60 days, with a simultaneous significant increase in alkaline phosphatase activity, further confirming the role of zinc in promoting bone formation [79].

Additionally, zinc maintains bone health by inhibiting the destruction of bone by osteoclasts. Osteoclasts are responsible for breaking down and removing old and damaged bone, and zinc can inhibit their activity, thereby maintaining an appropriate balance between old and new bone, known as the bone remodeling process. This balance is crucial for maintaining the integrity and stability of the bones [76,79].

#### 4.1.3. Copper

Copper stands as an indispensable trace element in bones, participating in the synthesis and stabilization of collagen, thereby influencing the structure and strength of bones. Research indicates that copper facilitates the activity of key enzymes in bones, further promoting the growth and differentiation of bone cells. Additionally, copper enhances the absorption and transport of calcium in bones, which is crucial for maintaining bone health and strength [80]. Therefore, copper is often incorporated into materials such as metallic titanium and bioceramics to enhance their osteogenic properties [81].

Firstly, copper promotes bone health by modulating inflammatory responses and oxidative stress. Studies have found that copper possesses anti-inflammatory and antioxidant properties, which are capable of reducing the damage caused to bones by inflammation and oxidative stress [80]. This mechanism aids in accelerating fracture healing and the treatment of bone diseases [82].

Secondly, copper enhances osteogenic activity by activating macrophages. Macrophages play a pivotal role in bone repair and regeneration. Research shows that copper ions can activate copper transport signals in macrophages, polarizing them into the pro-inflammatory M1 phenotype, thereby creating a favorable inflammatory microenvironment for the proliferation and differentiation of osteoblast-like cells [82].

Furthermore, copper can directly act on osteoblasts, promoting their proliferation and differentiation. Copper ions stimulate specific signaling pathways in osteoblasts, such as the BMP (bone morphogenetic protein) signaling pathway, thereby enhancing osteoblast activity and promoting the formation of new bone [83]. The BMP (bone morphogenetic protein) signaling pathway is a pivotal pathway that regulates cell differentiation, proliferation, and tissue morphogenesis, particularly in skeletal development and repair. Unlike the TGF-β pathway with which it shares SMAD effectors, BMP signaling specifically initiates upon ligand binding to type I/II serine/threonine kinase receptors, leading to the phosphorylation of receptor-regulated SMADs (R-SMADs: SMAD1/5/8). These then complex with SMAD4, translocate to the nucleus, and modulate target gene expression (e.g., Runx2, Id1). Its dysregulation is linked to bone disorders, vascular diseases, and cancer.

Lastly, copper also plays a significant role in bone remodeling. Bone remodeling is a dynamic process involving the resorption of old bone and the formation of new bone. By regulating this process, copper maintains a balance between old and new bone, thereby preserving the integrity and stability of bones [84].

### 4.2. Bioceramic Materials

#### 4.2.1. Hydroxyapatite

The chemical composition of hydroxyapatite (HAP) is similar to human bone tissue, consisting mainly of calcium and phosphorus, which are the most common components in bone tissue [85]. The calcium salts in bone tissue are primarily composed of crystalline apatite and amorphous calcium phosphate. Therefore, hydroxyapatite is not only compositionally similar but also structurally highly consistent with the calcium salts in human bone tissue. This similarity allows hydroxyapatite to integrate well with surrounding bone tissue after implantation, causing minimal rejection reactions and thereby facilitating bone tissue growth and repair [86].

Hydroxyapatite exhibits excellent bioactivity, capable of undergoing chemical reactions with surrounding tissues to induce differentiation of undifferentiated mesenchymal cells into osteoblasts and directly adhering to the surface of hydroxyapatite. This process promotes the adhesion, proliferation, and differentiation of bone cells, which is conducive to the formation of new bone. The porous structure of hydroxyapatite also provides a favorable environment for cell invasion and growth, further promoting bone tissue regeneration and repair [87].

The micron and nanometer structures of hydroxyapatite have a significant impact on its osteogenic properties. Studies have shown that both micron and nanometer structures on the surface of hydroxyapatite ceramics can influence the adhesion, proliferation, and differentiation of osteoblasts. Specifically, micron-patterned structures can promote cell adhesion, proliferation, and osteogenic differentiation, and as the size of the micron patterns decreases, the osteogenic differentiation ability of the cells increases. Nanostructures, such as nanorod structures, also demonstrate significant osteogenic capabilities. More importantly, combined micron-nanometer structures can synergistically promote cell adhesion, proliferation, and differentiation, exhibiting even more superior osteogenic effects [88].

Hydroxyapatite can also serve as a carrier for loading bone growth factors, such as bone morphogenetic protein-2 (BMP-2), enabling controlled release of the growth factor. BMP-2 is an important osteogenic inducer that promotes bone cell proliferation and differentiation. Through synergistic action with BMP-2, hydroxyapatite can significantly enhance osteogenic performance, promoting bone tissue regeneration and repair. For example, in silk fibroin-hydroxyapatite composite scaffolds, by optimizing the loading method of BMP-2 on the scaffold, active control of the controlled release behavior of BMP-2 can be achieved, thereby obtaining better osteogenic induction [88].

#### 4.2.2. Tricalcium Phosphate

β-Tricalcium phosphate (β-TCP) resembles the inorganic component of bone matrix and exhibits excellent biocompatibility and osteoinductivity. It can induce the formation of new bone in vivo, providing effective support for the repair of bone defects. This osteoinductivity may be related to its ability to promote the adhesion, proliferation, and differentiation of osteoblasts [89].

Tricalcium phosphate is often utilized as a scaffold material in bone tissue engineering. Its porous structure facilitates cell migration, growth, and vascularization, creating a favorable microenvironment for bone regeneration. In vivo, the tricalcium phosphate scaffold can guide the formation of new bone and gradually degrade to be replaced by new bone, thereby achieving the repair of bone defects. In a 2023 clinical trial, researchers treated patients with bone defects using a novel cotton-like bone graft substitute composed of β-tricalcium phosphate (β-TCP) and the bioabsorbable polymer poly(lactic-co-glycolic acid) (PLGA). All 48 cases achieved successful bone fusion, with an initial effectiveness rate of 100.0%. Postoperative CT scans at 24 weeks revealed a sustained effectiveness rate of 91.5%, demonstrating its excellent performance in promoting bone defect repair [90,91]. Recent research has increasingly focused on using tricalcium phosphate, which inherently favors bone formation, as a scaffold loaded with various novel substances to further enhance its osteogenic function, such as polylactic-co-glycolic acid (PLGA), curcumin compounds, and adipose-derived stem cell sheets [92,93].

Relevant studies have shown that tricalcium phosphate can interact with osteoblasts and other cells, affecting their biological behaviors. For instance, studies have inoculated osteoblast line MG-63 and primarily isolated and cultured rat bone marrow mesenchymal stem cells (rBMSCs) onto nanofibrous membrane materials with different β-TCP contents, finding that β-TCP can promote cell adhesion, proliferation, and osteogenic differentiation [93]. This may be due to β-TCP providing an appropriate surface microenvironment and chemical stimulation, thereby promoting osteogenic activity in cells.

Tricalcium phosphate also promotes osteogenesis by regulating cellular signaling pathways. Studies have indicated that β-TCP can activate the calcium-sensing receptor (CaSR) and bone morphogenetic protein (BMP)-2 signaling pathways, thereby inducing osteogenic differentiation of bone marrow mesenchymal stem cells (MSCs) [93]. The activation of these signaling pathways may promote the expression of osteogenic-related genes and the secretion of extracellular matrix, further facilitating the formation of new bone.

**Figure 4:**
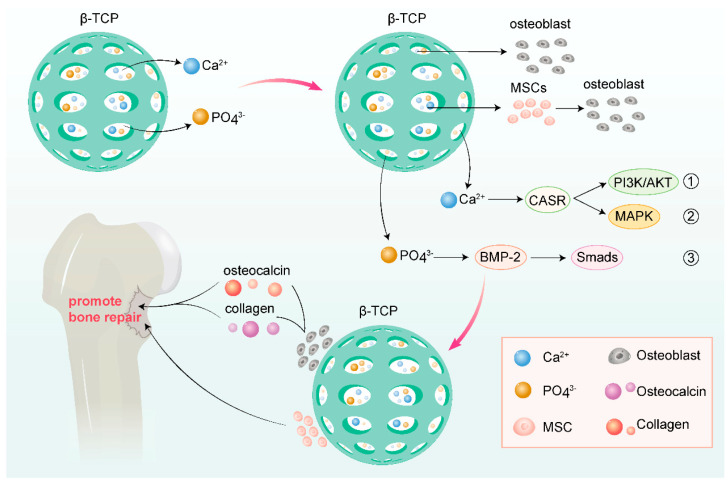
Various mechanisms of tricalcium phosphate promoting osteogenesis (drawn by Adobe Illustrator).

Another method to promote bone repair is through the transplantation of enriched bone marrow stem cells combined with tricalcium phosphate. Relevant research has shown that transplanting enriched bone marrow stem cells complexed with tricalcium phosphate to bone defect sites can significantly enhance osteogenic effects. This may be due to the self-renewal and osteogenic differentiation capabilities of stem cells, while tricalcium phosphate provides a favorable growth and differentiation environment for them [94] (Figure 4).

From the various materials with combined antimicrobial and osteogenic properties discussed above, it is evident that many promote osteogenesis, either by directly enhancing the BMP signaling pathway (e.g., copper ions and β-TCP) or by delivering core ligands of the BMP pathway, such as BMP-2, to stimulate bone formation (e.g., HAP). These shared mechanisms highlight that the BMP pathway serves as a common hub for osteogenic induction across diverse materials. Future designs should prioritize targeting key nodes of this pathway (e.g., receptor activation or SMAD stabilization) to optimize therapeutic outcomes.

## 5. Drug-Loading Capacity of Materials

### 5.1. Material Drug-Loading Requirements for Bone Infection

In the treatment of bone infections, there are multiple significant requirements for the drug-loading capacity of implanted biomaterials. Due to the difference in properties between bone infection sites and general infection sites, there are unique requirements for the drug-loading performance of the implanted biomaterials during the treatment of bone infections. When serving as drug carriers, biomaterials need to possess the ability to effectively load and slowly release relevant drugs to achieve good results in the treatment of implant-associated infections and promote bone tissue regeneration. For instance, Polylactic acid (PLA) and its copolymers exhibit excellent biocompatibility, meaning they do not elicit adverse reactions such as inflammation or rejection when in contact with biological tissues. This characteristic renders PLA and its copolymers as ideal materials for bone tissue engineering [95]. Similarly, Poly(glycolic acid) (PGA) and its copolymers possess excellent biocompatibility and biodegradability. They can gradually degrade in vivo and ultimately convert into water and carbon dioxide, which are harmless to the human body. This biodegradability has led to the widespread application of PGA and its copolymers in the biomedical field, particularly in fracture fixation and tissue engineering [96,97]. Good biocompatibility is just one aspect of the material’s drug-loading functionality. In addition, PLA and PGA themselves also have osteogenic properties. During their degradation process, they gradually release substances such as bone morphogenetic protein-2 (BMP-2) to regulate the expression of osteogenic-related genes in osteoblasts, providing a favorable environment for bone regeneration [98,99]. Their suitability for surface modification and other technological applications also gives them a unique advantage in terms of biological adaptability as drug delivery systems. These biomaterials, as drug carriers, play an active role in clearing bone infections and promoting bone regeneration. Therefore, in the treatment of bone infections, the implanted biomaterials not only need to possess good biocompatibility and mechanical properties but also need to have excellent drug-loading capacity and sustained-release properties to meet treatment requirements.

### 5.2. Design of Common Drug-Loaded Controlled Release Systems

#### 5.2.1. Drug-Loaded Bone Cement Systems

The bone cement drug delivery mechanism primarily involves loading antibiotics into polymethyl methacrylate (PMMA) bone cement for local drug delivery in orthopedic surgery. This system provides mechanical stability and is compatible with various heat-stable antibiotics. After the bone cement is implanted, it can release metabolically active antibacterial compounds above the minimum inhibitory concentration (MIC) of most common pathogens within a few hours to a few days. This not only eliminates the dead space or wound after debridement but also provides sustained antibiotic release to treat bone infections. However, the elution dynamics of this system vary widely, and incomplete antibiotic release may raise concerns about sustained low-level antibiotic release and subsequent antibiotic resistance [100].

#### 5.2.2. Bioactive Glass-Based Drug Delivery Systems

Bioglass materials possess bioactivity, enabling them to form a strong bond with bone tissue, which is attributed to the similarity of their chemical structure to the mineral phase of bone, thereby promoting bone conduction and osteoinductive properties. Furthermore, bioglass materials can serve as drug delivery carriers, utilizing their unique degradation properties to continuously and stably release antibacterial agents to combat bacterial infections while supporting bone growth. Additionally, this type of material has the ability to create a local environment unfavorable for microbial growth, which helps reduce the risk of infection and promotes healing of infected sites. To further enhance the performance of bioglass materials, combining type I collagen, mesoporous silica loaded with ciprofloxacin, and bioglass, and using molding methods to obtain scaffolds, not only improves the material’s ability to prevent the formation of bacterial biofilms and its antibacterial properties, but also achieves a breakthrough in enhancing the osteogenic properties of the material.

#### 5.2.3. Nanomaterial-Based Drug Delivery Systems

Nanomaterials, a recent research hotspot, have been widely applied in the past five years for their antibacterial and osteogenic properties, emerging as a popular drug delivery material for bone infections. The drug delivery role and mechanism of nanomaterials in bone infection treatment are mainly attributed to their unique physical and chemical properties, which enable them to effectively deliver drugs to the infection site while stimulating tissue regeneration and preventing bacterial infections.

Nanomaterials, with their minute size, can penetrate tissue gaps and cell membranes to achieve precise drug delivery. Through nanotechnology, drugs can be encapsulated within nanoparticles or adsorbed onto their surfaces, thereby enhancing drug stability and bioavailability. Simultaneously, nanomaterials can achieve sustained drug release by controlling their surface properties and structures to regulate the release rate and duration of drugs. This controllable release characteristic helps maintain effective drug concentrations at the infection site, reducing drug waste and side effects. Nanomaterials also exhibit excellent biocompatibility and bioactivity, with many of them being well-tolerated by biological tissues and exerting positive effects in vivo. For instance, nanostructured bioactive glasses can stimulate bone tissue regeneration and repair while creating a local environment unfavorable for microbial growth.

**Figure 5:**
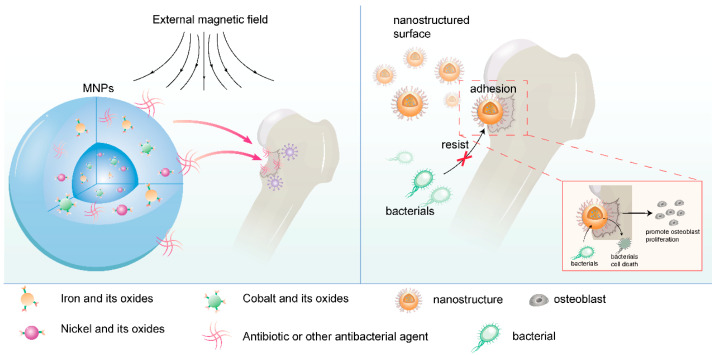
Nanomaterial drug delivery mechanism (drawn by Adobe Illustrator).

Common drug delivery mechanisms of nanomaterials include the following: ① Nanoparticle delivery systems: Based on magnetic nanoparticles (MNPs), which are typically composed of iron, nickel, cobalt, and their oxides, and can be manipulated through applied external magnetic fields. This system can directly deliver antibiotics and other antibacterial agents to the infection site and promote drug release and biofilm elimination through external magnetic stimulation. Nanoparticles can also be combined with other materials (such as polymers) to form multi-compartment polymeric formulations for more complex drug delivery strategies [101,102]. ② Nanopatterned surfaces: Nanopatterning refers to altering material properties by creating micrometer and nanometer-scale features on their surfaces [103]. These features can mimic natural textures, such as the nanocolumns on cicada wings or the nanotextures found on plants, lizards, and sharks. Through nanopatterning, surfaces with dual functions of antibacterial activity and tissue regeneration promotion can be created. Such surfaces can resist bacterial colonization and biofilm formation while promoting cell adhesion and proliferation [104,105] (Figure 5).

## 6. Challenges and Future Directions

Despite significant advancements in the research and development of medical biomaterials for bone infections, several challenges remain that need to be addressed to further enhance their efficacy and clinical application.

One of the primary challenges in the field is balancing the antibacterial efficacy of the materials with their biocompatibility. Many antibacterial materials, such as those containing metal ions or nanomaterials, exhibit potent antibacterial activity but may also have cytotoxic effects on surrounding tissues. Therefore, optimizing the material composition and surface properties to achieve both effective antibacterial action and minimal cytotoxicity is crucial. In the future, efforts could be made to develop smart responsive materials, such as those triggered by pH or enzymes, to release antimicrobial agents, enabling precise drug delivery at infection sites while reducing toxicity to normal tissues. Additionally, integrating the aforementioned surface modification techniques (e.g., biocompatible coatings) may further enhance the material’s biocompatibility.

The emergence of bacterial resistance to conventional antibiotics and even some advanced antibacterial materials poses a significant threat. This requires the continuous development of new antibacterial strategies and materials that can overcome bacterial resistance mechanisms, such as those based on novel antibacterial mechanisms or combination therapies. Multimodal antibacterial systems could also be explored, combining chemical antimicrobial strategies with physical methods (e.g., photodynamic therapy) to mitigate bacterial resistance risks. Furthermore, leveraging gene editing or high-throughput screening technologies could accelerate the discovery of novel antibacterial targets, providing new insights for material design.

While many medical biomaterials focus on antibacterial properties, it is equally important to enhance their osteogenic properties to promote bone regeneration and repair. This involves optimizing the material’s surface topography, porosity, and chemistry to support cell adhesion, proliferation, and differentiation into osteoblasts. Building on the materials mentioned in this review that exhibit both antibacterial and osteogenic properties, further research and development could focus on materials with similar performance characteristics.

Ensuring the long-term stability and durability of medical biomaterials in vivo is another challenge. Materials must be able to withstand the mechanical forces and biological environments present in the body without degrading prematurely or losing their functional properties. Future solutions may involve creating composite materials with controlled degradation rates; for instance, by adjusting the degradation kinetics of polymers or ceramics. Additionally, incorporating self-healing material technologies or dynamic crosslinking strategies could enhance mechanical properties and long-term stability, thereby extending the functional lifespan of these materials in vivo.

In addition, despite the remarkable antibacterial efficacy demonstrated by metal ions (e.g., Ag⁺, Sr^2^⁺, Zn^2^⁺) and nanomaterials (e.g., AgNPs, ZnO NPs) in anti-bone infection applications, their metabolic pathways and potential accumulation issues in vivo warrant significant attention. Studies have shown that these materials may distribute to organs, such as the liver, spleen, and kidneys through blood circulation, with prolonged retention potentially inducing oxidative stress or inflammatory responses [67,68] For instance, while silver ions can be excreted via bile and urine, high-dose administration may still lead to local tissue deposition. Certain non-degradable nanomaterials (e.g., TiO_2_ NPs) may persist in the lymphatic system or bone marrow over extended periods. Furthermore, the release kinetics of metal ions are closely linked to material degradation behavior, with improper release rates exacerbating systemic exposure risks. To address these challenges, future research should focus on developing intelligent material systems capable of precisely regulating release behaviors. Additionally, establishing robust in vivo tracking technologies and long-term toxicity evaluation systems will be pivotal in ensuring the safe clinical application of these materials (Table 1).

In addition to the aforementioned strategies addressing the difficulties and challenges in the application of various biomaterials identified during our research, future development directions for medical biomaterials in bone infection treatment should also focus on the following aspects.

The development of multifunctional materials that combine antibacterial, osteogenic, and other desired properties (such as angiogenesis or immunomodulation) is a promising future direction. This would enable the treatment of bone infections while simultaneously promoting bone regeneration and reducing the risk of implant failure.

The use of advanced characterization techniques, such as high-resolution imaging, spectroscopy, and molecular biology tools, will be crucial for understanding the interactions between medical biomaterials and the biological environment. These techniques can provide insights into the mechanisms of action of antibacterial materials, the response of cells to osteogenic cues, and the degradation behavior of implants in vivo.

The integration of personalized medicine approaches, including genetic profiling and patient-specific tissue engineering, could lead to the development of medical biomaterials that are tailored to the individual patient’s needs. This would enhance the efficacy and safety of the materials, as well as improve patient outcomes.

Collaborative research efforts between materials scientists, biologists, clinicians, and engineers will be essential for overcoming the challenges in this field. By combining expertise from different disciplines, it will be possible to develop innovative medical biomaterials that address the complex needs of patients with bone infections.

In conclusion, while significant progress has been made in the research of medical biomaterials for bone infections, several challenges remain. By addressing these challenges and pursuing future directions, such as multifunctional materials, advanced characterization techniques, personalized medicine approaches, and collaborative research efforts, it is possible to further enhance the efficacy and clinical application of these materials.

## 7. Conclusions

In this review, we have comprehensively examined the recent research progress in the field of medical biomaterials for the treatment of bone infections, encompassing a diverse array of materials with both antibacterial and osteogenic properties. Specifically, we discussed antimicrobial metals and alloys (including silver ions, strontium ions, cobalt ions, etc.), nanomaterials (such as nano-zinc oxide and nano-titanium dioxide), antimicrobial peptides (like LL-37, cecropins, and melittin), enzymes (such as lysozyme and glucose oxidase), polysaccharides (including chitosan and sodium alginate), and antibacterial materials with osteogenic potential (such as magnesium, zinc, copper ions and their compounds, hydroxyapatite, tricalcium phosphate, etc.). These materials offer a variety of options for the treatment of bone infections through distinct antibacterial mechanisms and osteogenic pathways.

During the research and development of biomaterials for bone infections, particular emphasis should be placed on balancing the antibacterial performance and biocompatibility of these materials. Ideal biomaterials should not only possess efficient antibacterial capabilities to effectively eradicate infectious bacteria and prevent infection recurrence but also exhibit good biocompatibility to avoid inducing toxic effects or triggering immune responses in surrounding tissues. Additionally, the degradation performance and drug release characteristics of the materials are crucial areas of study. Reasonable degradation rates ensure that the materials are gradually absorbed or excreted by the body after fulfilling their therapeutic roles, while sustained and stable drug release helps maintain effective drug concentrations at the treatment site, thereby enhancing therapeutic efficacy. Simultaneously, adverse reactions induced by the materials, such as allergic reactions, inflammatory responses, or long-term toxic accumulation, should be minimized to ensure their safety in clinical applications.

The application of medical biomaterials for bone infections holds significant implications for patients suffering from bone infections. With ongoing research and technological advancements, it is anticipated that more efficient and safe medical biomaterials will be developed in the future, providing more effective treatment options for bone infection patients and further improving their prognosis and quality of life.

## Figures and Tables

**Table 1:** List of antibacterial materials for bone infection.

Material	Mechanism	Function	Application
Silver ion (Ag^+^)	Interact with phospholipids and sulfhydryl groups (-SH) in bacterial cell membranes to destroy cell membrane structure and function [16] Interacts with proteins and DNA to inhibit bacterial growth [13] Induces the production of reactive oxygen species and causes oxidative stress	Antisepsis; anti-biofilm formation	Dressing eptic; antibacterial medical device (e.g., catheter, cardiac valves) [19]
Silver nanoparticle (Ag NPs)	Penetrates the bacterial cell wall and membrane, and acts directly on the inside of the bacteria [16] Inhibit biofilm formation [16]	Antisepsis; anti-biofilm formation	Treating chronic infections; treating implant-associated infections [19]
Strontium ion (Sr)	Damages the bacterial cell membrane structure and increases permeability [22] Inhibits bacterial biofilm formation [22] Activates the host immune response and promotes macrophage polarization [23]	Antisepsis; promotes bone formation; inhibits bone resorption	Bone regeneration materials; [23] antibacterial implants
Cobalt ions (Co)	Infiltrated into bacterial cells, damaging DNA, RNA, and enzymes [25] Affects the permeability of the cell membrane [25] Combines with other materials to form composites, continuously releasing cobalt ions [26]	Antisepsis; promotes bone repair	Fracture fixation implants; bone defect repair materials [27]
Nano-zinc oxide (ZnO NPs)	Acts directly with bacterial cell membranes, destroying the membrane structure Releases Zn^2+^ ions, causing oxidative stress [36] Inhibits protein and DNA synthesis [39] Activates the perennial immune system [40]	Antisepsis; anti-biofilm formation	Antiseptic paint; medical apparatus
Nanometer titania (TiO_2_NPs)	They generate reactive oxygen species (ROS) under light and destroy bacterial cell membranes [44] Interferes with bacterial energy transport and protein function [45]	Antisepsis; anti-biofilm formation	Bone infection treatment; antibacterial coating
Antibacterial peptide (LL-37)	Binds to lipoteichoic acid or LPS on the bacterial cell wall and destroys the cell wall [46] Inhibits biofilm formation [48] Modulates an immune response Promotes wound healing	Antisepsis; anti-biofilm formation; immunoregulation	Antimicrobial dressings; immunomodulator
Sericin antimicrobial peptide	Forms a spiral structure of two parts, destroys the bacterial cell membrane [50] Forms ion penetration channels, leading to cell depolarization [50] Inhibits bacterial metabolic activities	Antisepsis; anti-biofilm formation	Antibacterial agents; anti-infective therapy
Melittin	Non-specific binding to bacterial cell membrane to damage membrane structure; [58] Inhibits cell metabolism Induces bacterial autolysis	Antisepsis; antifungal; antiviral; antitumor	Antibacterial agents; antitumor therapy [55]
Lysozyme	Lyses peptidoglycan in bacterial cell walls (Gram-positive bacteria) [58] Synergizes with other antimicrobials to enhance Gram-negative inhibition Regulates the inflammatory microenvironment via cerium ions’ antioxidant activity [59]	Antisepsis; anti-biofilm formation; promotes bone regeneration	Infected bone defect repair; sequential drug delivery systems
Glucose Oxidase (GOx)	Catalyzes β-D-glucose oxidation to gluconic acid and H_2_O_2_, depleting bacterial energy [60] Generates hydroxyl radicals (•OH) via Fenton-like reactions with metal ions [60] Polarizes macrophages to the M2 anti-inflammatory phenotype	Antisepsis; metabolic intervention; immunomodulation	Diabetic bone infection therapy; photo-enhanced chemodynamic therapy
Chitosan (CS)	Electrostatic interaction with bacterial membranes [64] Binds DNA and chelates Mg^2^⁺/Ca^2^⁺, disrupting metabolism [64] Molecular weight-dependent action (high-MW: Gram+; low-MW: Gram−)	Antisepsis; anti-biofilm formation; promotes bone formation	Infected bone defect repair; 3D-printed scaffolds with osteogenic/antibacterial dual functions
Sodium Alginate (SA)	Electrostatic interaction with bacterial membranes (carboxyl groups) pH-responsive activity (pH 5.5–6.5 targets inflammatory sites) Chelates Ca^2^⁺/Zn^2^⁺ to form osteogenic hydrogels [67]	Antisepsis; drug delivery carrier; promotes vascularization and mineralization [70]	Bone infection management; composite hydrogels with antibiotics/nanoparticles
Magnesium (Mg)	Magnesium ion activates the Pl3K/Akt signaling pathway and promotes osteoblast proliferation and differentiation [74,75] Disrupts the integrity of bacterial cell membranes	Antisepsis; promotes bone formation	Bone graft materials; fracture repair
Zinc (Zn)	Stimulates osteoblast proliferation and differentiation [76] Enhances alkaline phosphatase activity [79] Inhibits osteoclast activity	Antisepsis; promotes bone formation	Bone repair material; osteoporosis therapy
Copper (Cu)	Regulates the inflammatory response and oxidative stress [80] Activates macrophages to promote osteogenic activity [82] Cu acts directly on osteoblasts to promote proliferation and differentiation [83]	Antisepsis; promotes bone formation	Bone repair materials; fracture healing
Hydroxyapatite (HAP)	Similar to bone tissue composition [87] Promotes bone cell adhesion, proliferation, and differentiation [88] Acts as a bone growth factor carrier to promote bone regeneration [86]	Antisepsis; promotes bone formation	Bone tissue engineering; bone defect repair
Tertiary calcium phosphate (β-TCP)	Provides a suitable microenvironment to promote cell adhesion, proliferation, and differentiation [93] Activates calcium-sensing receptor (CaSR) and BMP-2 signaling pathways to promote osteogenic differentiation [93]	Antisepsis; promotes bone formation	Bone tissue engineering; bone defect repair

## Data Availability

No new data and not applicable.

## References

[1] Geurts J.A.P., van Vugt T.A.G., Arts J.J.C. (2021). Use of contemporary biomaterials in chronic osteomyelitis treatment: Clinical lessons learned and literature review. J. Orthop. Res..

[2] Sendi P., Lora-Tamayo J., Cortes-Penfield N.W., Uçkay I. (2023). Early switch from intravenous to oral antibiotic treatment in bone and joint infections. Clin. Microbiol. Infect..

[3] Masters E.A., Ricciardi B.F., Bentley K.L.d.M., Moriarty T.F., Schwarz E.M., Muthukrishnan G. (2022). Skeletal infections: Microbial pathogenesis, immunity and clinical management. Nat. Rev. Microbiol..

[4] Zhu W., Li C., Yao M., Wang X., Wang J., Zhang W., Chen W., Lv H. (2023). Advances in osseointegration of biomimetic mineralized collagen and inorganic metal elements of natural bone for bone repair. Regen. Biomater..

[5] Shuaishuai W., Tongtong Z., Dapeng W., Mingran Z., Xukai W., Yue Y., Hengliang D., Guangzhi W., Minglei Z. (2023). Implantable biomedical materials for treatment of bone infection. Front. Bioeng. Biotechnol..

[6] Gibb B.P., Hadjiargyrou M. (2021). Bacteriophage therapy for bone and joint infections. Bone Jt. J..

[7] Tsegka K.G., Voulgaris G.L., Kyriakidou M., Kapaskelis A., Falagas M.E. (2022). Intravenous fosfomycin for the treatment of patients with bone and joint infections: A review. Expert Rev. Anti-Infect. Ther..

[8] Wang C., Xu P., Li X., Zheng Y., Song Z. (2022). Research progress of stimulus-responsive antibacterial materials for bone infection. Front. Bioeng. Biotechnol..

[9] Hofmann S.R., Kapplusch F., Girschick H.J., Morbach H., Pablik J., Ferguson P.J., Hedrich C.M. (2017). Chronic Recurrent Multifocal Osteomyelitis (CRMO): Presentation, Pathogenesis, and Treatment. Curr. Osteoporos. Rep..

[10] Nikolova M.P., Apostolova M.D. (2022). Apostolova, Advances in Multifunctional Bioactive Coatings for Metallic Bone Implants. Materials.

[11] Jiao J., Zhang S., Qu X., Yue B. (2021). Recent Advances in Research on Antibacterial Metals and Alloys as Implant Materials. Front. Cell. Infect. Microbiol..

[12] Wang Y., Jan H., Zhong Z., Zhou L., Teng K., Chen Y., Xu J., Xie D., Chen D., Xu J. (2025). Multiscale metal-based nanocomposites for bone and joint disease therapies. Mater. Today Bio.

[13] Choi Y.S., Kim Y.H., An H.M., Bae S.K., Lee Y.K. (2023). Efficacy of Silver Nanoparticles-Loaded Bone Cement against an MRSA Induced-Osteomyelitis in a Rat Model. Medicina.

[14] Żyro D., Sikora J., Szynkowska-Jóźwik M.I., Ochocki J. (2023). Silver, Its Salts and Application in Medicine and Pharmacy. Int. J. Mol. Sci..

[15] Kędziora A., Speruda M., Krzyżewska E., Rybka J., Łukowiak A., Bugla-Płoskońska G. (2018). Similarities and Differences Between Silver Ions and Silver in Nanoforms as Antibacterial Agents. Int. J. Mol. Sci..

[16] Soma T., Iwasaki R., Sato Y., Kobayashi T., Ito E., Matsumoto T., Kimura A., Homma F., Saiki K., Takahashi Y. (2022). An ionic silver coating prevents implant-associated infection by anaerobic bacteria in vitro and in vivo in mice. Sci. Rep..

[17] Hu C.C., Chang C.H., Chang Y., Hsieh J.H., Ueng S.W. (2020). Beneficial Effect of TaON-Ag Nanocomposite Titanium on Antibacterial Capacity in Orthopedic Application. Int. J. Nanomed..

[18] Kose N., Otuzbir A., Pekşen C., Kiremitçi A., Doğan A. (2013). A silver ion-doped calcium phosphate-based ceramic nanopowder-coated prosthesis increased infection resistance. Clin. Orthop. Relat. Res..

[19] Funao H., Nagai S., Sasaki A., Hoshikawa T., Tsuji T., Okada Y., Koyasu S., Toyama Y., Nakamura M., Aizawa M. (2016). A novel hydroxyapatite film coated with ionic silver via inositol hexaphosphate chelation prevents implant-associated infection. Sci. Rep..

[20] Kheirmand-Parizi M., Doll-Nikutta K., Gaikwad A., Denis H., Stiesch M. (2024). Effectiveness of strontium/silver-based titanium surface coatings in improving antibacterial and osteogenic implant characteristics: A systematic review of in-vitro studies. Front. Bioeng. Biotechnol..

[21] Silva A.V., Gomes D.d.S., Victor R.d.S., Santana L.N.d.L., Neves G.A., Menezes R.R. (2023). Influence of Strontium on the Biological Behavior of Bioactive Glasses for Bone Regeneration. Materials.

[22] Li Y., Wang W., Han J., Li Z., Wang Q., Lin X., Ge K., Zhou G. (2022). Synthesis of Silver- and Strontium-Substituted Hydroxyapatite with Combined Osteogenic and Antibacterial Activities. Biol. Trace. Elem. Res..

[23] Parizi M.K., Doll K., Rahim M.I., Mikolai C., Winkel A., Stiesch M. (2022). Antibacterial and Cytocompatible: Combining Silver Nitrate with Strontium Acetate Increases the Therapeutic Window. Int. J. Mol. Sci..

[24] Jia C., Wu F.G. (2023). Antibacterial Chemodynamic Therapy: Materials and Strategies. BME Front..

[25] Watanabe K., Fukuzaki S., Sugino A., Benson N., Metcalf N., Nakamura M., Matsumoto M. (2021). Cobalt-Chromium Alloy Has Superior Antibacterial Effect Than Titanium Alloy: In Vitro and In Vivo Studies. Spine.

[26] Khalid A., Ahmad P., Alharthi A.I., Muhammad S., Khandaker M.U., Faruque M.R.I., Khan A., Din I.U., Alotaibi M.A., Alzimami K. (2021). Enhanced Optical and Antibacterial Activity of Hydrothermally Synthesized Cobalt-Doped Zinc Oxide Cylindrical Microcrystals. Materials.

[27] Kim D., Park K.W., Park J.T., Choi I. (2023). Photoactive MOF-Derived Bimetallic Silver and Cobalt Nanocomposite with Enhanced Antibacterial Activity. ACS Appl. Mater. Interfaces.

[28] Vila J., Moreno-Morales J., Ballesté-Delpierre C. (2020). Current landscape in the discovery of novel antibacterial agents. Clin. Microbiol. Infect..

[29] Zheng Y., Wei M., Wu H., Li F., Ling D. (2022). Antibacterial metal nanoclusters. J. Nanobiotechnol..

[30] Wang B., Xu Y., Shao D., Li L., Ma Y., Li Y., Zhu J., Shi X., Li W. (2022). Inorganic nanomaterials for intelligent photothermal antibacterial applications. Front. Bioeng. Biotechnol..

[31] Fan S., Lin W., Huang Y., Xia J., Xu J.F., Zhang J., Pi J. (2022). Advances and Potentials of Polydopamine Nanosystem in Photothermal-Based Antibacterial Infection Therapies. Front. Pharmacol..

[32] Asadi L., Mokhtari J., Abbasi M. (2021). An alginate-PHMB-AgNPs based wound dressing polyamide nanocomposite with improved antibacterial and hemostatic properties. J. Mater. Sci. Mater. Med..

[33] Bee S.L., Bustami Y., Ul-Hamid A., Lim K., Abdul Hamid Z.A. (2021). Synthesis of silver nanoparticle-decorated hydroxyapatite nanocomposite with combined bioactivity and antibacterial properties. J. Mater. Sci. Mater. Med..

[34] Lagana A., Visalli G., Corpina F., Ferlazzo M., Di Pietro A., Facciola A. (2023). Antibacterial activity of nanoparticles and nanomaterials: A possible weapon in the fight against healthcare-associated infections. Eur. Rev. Med. Pharmacol. Sci..

[35] Li Y., Yang Y., Qing Y., Li R., Tang X., Guo D., Qin Y. (2020). Enhancing ZnO-NP Antibacterial and Osteogenesis Properties in Orthopedic Applications: A Review. Int. J. Nanomed..

[36] Mendes C.R., Dilarri G., Forsan C.F., Sapata V.M.R., Lopes P.R.M., de Moraes P.B., Montagnolli R.N., Ferreira H., Bidoia E.D. (2022). Antibacterial action and target mechanisms of zinc oxide nanoparticles against bacterial pathogens. Sci. Rep..

[37] Thapliyal D., Verros G.D., Arya R.K. (2025). Nanoparticle-Doped Antibacterial and Antifungal Coatings. Polymers.

[38] Mutukwa D., Taziwa R.T., Khotseng L. (2024). A Review of Plant-Mediated ZnO Nanoparticles for Photodegradation and Antibacterial Applications. Nanomaterials.

[39] Hammad S.M., El-Wassefy N.A., Shamaa M.S., Fathy A. (2020). Evaluation of zinc-oxide nanocoating on the characteristics and antibacterial behavior of nickel-titanium alloy. Dental. Press J. Orthod..

[40] Ye K., Huang M., He X., An Z., Qin H. (2022). Synergistic Antibacterial Effect of Zinc Oxide Nanoparticles and Polymorphonuclear Neutrophils. J. Funct. Biomater..

[41] Okaiyeto K., Gigliobianco M.R., Di Martino P. (2024). Biogenic Zinc Oxide Nanoparticles as a Promising Antibacterial Agent: Synthesis and Characterization. Int. J. Mol. Sci..

[42] Missier M.S., Ramakrishnan M., Sudalaimani Paulpandian S.D., Rajeshkumar S., Tania M. (2023). Antibacterial, antioxidant and anti-inflammatory activity zinc-titanium dioxide nanocomposite. Bioinformation.

[43] Habib S., Rashid F., Tahir H., Liaqat I., Latif A.A., Naseem S., Khalid A., Haider N., Hani U., Dawoud R.A. (2023). Antibacterial and Cytotoxic Effects of Biosynthesized Zinc Oxide and Titanium Dioxide Nanoparticles. Microorganisms.

[44] Rothpan M., Chandra Teja Dadi N., McKay G., Tanzer M., Nguyen D., Hart A., Tabrizian M. (2023). Titanium-Dioxide-Nanoparticle-Embedded Polyelectrolyte Multilayer as an Osteoconductive and Antimicrobial Surface Coating. Materials.

[45] Pangprasit N., Thammawong Y., Kulsirorat A., Chuammitri P., Kongkaew A., Intanon M., Suriyasathaporn W., Pikulkaew S., Chaisri W. (2023). Titanium Dioxide Nano-Formulation: Characterization, Antimicrobial Activity, and Wound Healing in Animals. Animals.

[46] Zhuo H., Zhang X., Li M., Zhang Q., Wang Y. (2022). Antibacterial and Anti-Inflammatory Properties of a Novel Antimicrobial Peptide Derived from LL-37. Antibiotics.

[47] Dennison S.R., Reddy S.M., Morton L.H.G., Harris F., Badiani K., Phoenix D.A. (2022). PEGylation enhances the antibacterial and therapeutic potential of amphibian host defence peptides. Biochim. Biophys. Acta Biomembr..

[48] Ridyard K.E., Overhage J. (2021). The Potential of Human Peptide LL-37 as an Antimicrobial and Anti-Biofilm Agent. Antibiotics.

[49] Nagaoka I., Tamura H., Reich J. (2020). Therapeutic Potential of Cathelicidin Peptide LL-37, an Antimicrobial Agent, in a Murine Sepsis Model. Int. J. Mol. Sci..

[50] Henao Arias D.C., Toro L.J., Téllez Ramirez G.A., Osorio-Méndez J.F., Rodríguez-Carlos A., Valle J., Marín-Luevano S.P., Rivas-Santiago B., Andreu D., Castaño Osorio J.C. (2021). Novel antimicrobial cecropins derived from O. curvicornis and D. satanas dung beetles. Peptides.

[51] Tian Y., Wei H., Lu F., Wu H., Lou D., Wang S., Geng T. (2024). Antibacterial mechanism and structure-activity relationships of Bombyx mori cecropin A. Insect Mol. Biol..

[52] Ramos-Martín F., Herrera-León C., D’Amelio N. (2022). Molecular basis of the anticancer, apoptotic and antibacterial activities of Bombyx mori Cecropin A. Arch. Biochem. Biophys..

[53] Wojda I., Cytryńska M., Zdybicka-Barabas A., Kordaczuk J. (2020). Insect Defense Proteins and Peptides. Subcell. Biochem..

[54] Askari P., Namaei M.H., Ghazvini K., Hosseini M. (2021). In vitro and in vivo toxicity and antibacterial efficacy of melittin against clinical extensively drug-resistant bacteria. BMC Pharmacol. Toxicol..

[55] Xu X., Fu H., Wu W., Zong L., Li D., Zhuang B., Qi Y., Qi X., Liang T. (2025). Synthesis and Evaluation of Melittin-Modified Peptides for Antibacterial Activity. Toxins.

[56] Nawaz N., Wen S., Wang F., Nawaz S., Raza J., Iftikhar M., Usman M. (2022). Lysozyme and Its Application as Antibacterial Agent in Food Industry. Molecules.

[57] Zhao M., Huang M., Li Z. (2023). Exploring the therapeutic potential of recombinant human lysozyme: A review on wound management system with antibacterial. Front. Bioeng. Biotechnol..

[58] Monavari M., Medhekar R., Nawaz Q., Monavari M., Fuentes-Chandía M., Homaeigohar S., Boccaccini A.R. (2022). A 3D Printed Bone Tissue Engineering Scaffold Composed of Alginate Dialdehyde-Gelatine Reinforced by Lysozyme Loaded Cerium Doped Mesoporous Silica-Calcia Nanoparticles. Macromol. Biosci..

[59] Shen L., Cao S., Wang Y., Zhou P., Wang S., Zhao Y., Meng L., Zhang Q., Li Y., Xu X. (2023). Self-Adaptive Antibacterial Scaffold with Programmed Delivery of Osteogenic Peptide and Lysozyme for Infected Bone Defect Treatment. ACS Appl. Mater. Interfaces.

[60] He M., Wang H., Han Q., Shi X., He S., Sun J., Zhu Z., Gan X., Deng Y. (2023). Glucose-primed PEEK orthopedic implants for antibacterial therapy and safeguarding diabetic osseointegration. Biomaterials.

[61] Liao Y., Zhang Z., Zhao Y., Zhang S., Zha K., Ouyang L., Hu W., Zhou W., Sun Y., Liu G. (2025). Glucose oxidase: An emerging multidimensional treatment option for diabetic wound healing. Bioact. Mater..

[62] Tian Y., Wu D., Wu D., Cui Y., Ren G., Wang Y., Wang J., Peng C. (2022). Chitosan-Based Biomaterial Scaffolds for the Repair of Infected Bone Defects. Front. Bioeng. Biotechnol..

[63] Egorov A.R., Kirichuk A.A., Rubanik V.V., Tskhovrebov A.G., Kritchenkov A.S. (2023). Chitosan and Its Derivatives: Preparation and Antibacterial Properties. Materials.

[64] Zhang J., Ye X., Li W., Lin Z., Wang W., Chen L., Li Q., Xie X., Xu X., Lu Y. (2023). Copper-containing chitosan-based hydrogels enabled 3D-printed scaffolds to accelerate bone repair and eliminate MRSA-related infection. Int. J. Biol. Macromol..

[65] Yu D., Feng J., You H., Zhou S., Bai Y., He J., Cao H., Che Q., Guo J., Su Z. (2022). The Microstructure, Antibacterial and Antitumor Activities of Chitosan Oligosaccharides and Derivatives. Mar. Drugs.

[66] Zhang H., Cheng J., Ao Q. (2021). Preparation of Alginate-Based Biomaterials and Their Applications in Biomedicine. Mar. Drugs.

[67] Wu X.X., Zhang Y., Hu T., Li W.X., Li Z.L., Hu H.J., Zhu S.R., Chen W.Z., Zhou C.S., Jiang G.B. (2021). Long-term antibacterial composite via alginate aerogel sustained release of antibiotics and Cu used for bone tissue bacteria infection. Int. J. Biol. Macromol..

[68] He Q., Tong T., Yu C., Wang Q. (2022). Advances in Algin and Alginate-Hybrid Materials for Drug Delivery and Tissue Engineering. Mar. Drugs.

[69] Wathoni N., Herdiana Y., Suhandi C., Mohammed A.F.A., El-Rayyes A., Narsa A.C. (2024). Chitosan/Alginate-Based Nanoparticles for Antibacterial Agents Delivery. Int. J. Nanomed..

[70] Yuan N., Shao K., Huang S., Chen C. (2023). Chitosan, alginate, hyaluronic acid and other novel multifunctional hydrogel dressings for wound healing: A review. Int. J. Biol. Macromol..

[71] He X., Li Y., Zou D., Zu H., Li W., Zheng Y. (2024). An overview of magnesium-based implants in orthopaedics and a prospect of its application in spine fusion. Bioact. Mater..

[72] Azadani M.N., Zahedi A., Bowoto O.K., Oladapo B.I. (2022). A review of current challenges and prospects of magnesium and its alloy for bone implant applications. Prog. Biomater..

[73] Kang Y., Xu C., Meng L., Dong X., Qi M., Jiang D. (2022). Exosome-functionalized magnesium-organic framework-based scaffolds with osteogenic, angiogenic and anti-inflammatory properties for accelerated bone regeneration. Bioact. Mater..

[74] Hu J., Shao J., Huang G., Zhang J., Pan S. (2023). In Vitro and In Vivo Applications of Magnesium-Enriched Biomaterials for Vascularized Osteogenesis in Bone Tissue Engineering: A Review of Literature. J. Funct. Biomater..

[75] Gong C., Yang J., Zhang X., Wang X., Wei Z., Huang X., Guo W. (2024). Surface functionalization of calcium magnesium phosphate cements with alginate sodium for enhanced bone regeneration via TRPM7/PI3K/Akt signaling pathway. Int. J. Biol. Macromol..

[76] Chen Z., Zhang W., Wang M., Backman L.J., Chen J. (2022). Effects of Zinc, Magnesium, and Iron Ions on Bone Tissue Engineering. ACS Biomater. Sci. Eng..

[77] O’connor J.P., Kanjilal D., Teitelbaum M., Lin S.S., Cottrell J.A. (2020). Zinc as a Therapeutic Agent in Bone Regeneration. Materials.

[78] Wang S., Li R., Xia D., Zhao X., Zhu Y., Gu R., Yoon J., Liu Y. (2021). The impact of Zn-doped synthetic polymer materials on bone regeneration: A systematic review. Stem Cell Res. Ther..

[79] Molenda M., Kolmas J. (2023). The Role of Zinc in Bone Tissue Health and Regeneration-a Review. Biol. Trace. Elem. Res..

[80] Noori A., Hoseinpour M., Kolivand S., Lotfibakhshaiesh N., Azami M., Ai J., Ebrahimi-Barough S. (2022). Synergy effects of copper and L-arginine on osteogenic, angiogenic, and antibacterial activities. Tissue Cell.

[81] Šalandová M., van Hengel I.A.J., Apachitei I., Zadpoor A.A., van der Eerden B.C.J., Fratila-Apachitei L.E. (2021). Inorganic Agents for Enhanced Angiogenesis of Orthopedic Biomaterials. Adv. Heal. Mater..

[82] Wang Y., Zhang W., Yao Q. (2021). Copper-based biomaterials for bone and cartilage tissue engineering. J. Orthop. Translat..

[83] Yan F., Lv M., Zhang T., Zhang Q., Chen Y., Liu Z., Wei R., Cai L. (2021). Copper-Loaded Biodegradable Bone Wax with Antibacterial and Angiogenic Properties in Early Bone Repair. ACS Biomater. Sci. Eng..

[84] Zhang Z., Tang H., Du T., Yang D. (2024). The impact of copper on bone metabolism. J. Orthop. Transl..

[85] Ran L., Liu L., Gao J., Pan Y., Ramalingam M., Du X., Liu Y., Cheng L., Shi Z. (2023). Strontium-doped hydroxyapatite and its role in osteogenesis and angiogenesis. Int. J. Dev. Biol..

[86] Hiromoto S., Nozoe E., Hanada K., Yoshimura T., Shima K., Kibe T., Nakamura N., Doi K. (2021). In vivo degradation and bone formation behaviors of hydroxyapatite-coated Mg alloys in rat femur. Mater. Sci. Eng. C Mater. Biol. Appl..

[87] Fitzpatrick V., Martín-Moldes Z., Deck A., Torres-Sanchez R., Valat A., Cairns D., Li C., Kaplan D.L. (2021). Functionalized 3D-printed silk-hydroxyapatite scaffolds for enhanced bone regeneration with innervation and vascularization. Biomaterials.

[88] Liang W., Ding P., Li G., Lu E., Zhao Z. (2021). Hydroxyapatite Nanoparticles Facilitate Osteoblast Differentiation and Bone Formation Within Sagittal Suture During Expansion in Rats. Drug Des. Devel. Ther..

[89] Funayama T., Noguchi H., Kumagai H., Sato K., Yoshioka T., Yamazaki M. (2021). Unidirectional porous beta-tricalcium phosphate and hydroxyapatite artificial bone: A review of experimental evaluations and clinical applications. J. Artif. Organs..

[90] Jeong J.H., Jin E.S., Kim J.Y., Min J., Jeon S.R., Lee M., Choi K.H. (2022). Bone formation effect of highly concentrated tricalcium phosphate biocomposite screws in a rabbit osteoporosis model. J. Orthop. Res..

[91] Sachse A., Hasenbein I., Hortschansky P., Schmuck K.D., Maenz S., Illerhaus B., Kuehmstedt P., Ramm R., Huber R., Kunisch E. (2023). BMP-2 (and partially GDF-5) coating significantly accelerates and augments bone formation close to hydroxyapatite/tricalcium-phosphate/brushite implant cylinders for tibial bone defects in senile, osteopenic sheep. J. Mater. Sci. Mater. Med..

[92] Uchikawa E., Yoshizawa M., Li X., Matsumura N., Li N., Chen K., Kagami H. (2021). Tooth transplantation with a β-tricalcium phosphate scaffold accelerates bone formation and periodontal tissue regeneration. Oral. Dis..

[93] Wei S., Zhang R.G., Wang Z.Y. (2022). Deferoxamine/magnesium modified β-tricalcium phosphate promotes the bone regeneration in osteoporotic rats. J. Biomater. Appl..

[94] Ye X., Zhang Y., Liu T., Chen Z., Chen W., Wu Z., Wang Y., Li J., Li C., Jiang T. (2022). Beta-tricalcium phosphate enhanced mechanical and biological properties of 3D-printed polyhydroxyalkanoates scaffold for bone tissue engineering. Int. J. Biol. Macromol..

[95] Zheng Z., Wang R., Lin J., Tian J., Zhou C., Li N., Li L. (2022). Liquid Crystal Modified Polylactic Acid Improves Cytocompatibility and M2 Polarization of Macrophages to Promote Osteogenesis. Front. Bioeng. Biotechnol..

[96] Chen L., Tian M., Yang J., Wu Z. (2023). Berberine-Encapsulated Poly(lactic-co-glycolic acid)-Hydroxyapatite (PLGA/HA) Microspheres Synergistically Promote Bone Regeneration with DOPA-IGF-1 via the IGF-1R/PI3K/AKT/mTOR Pathway. Int. J. Mol. Sci..

[97] Xie X., Shi X., Wang S., Cao L., Yang C., Ma Z. (2020). Effect of Attapulgite-Doped Electrospun Fibrous PLGA Scaffold on Pro-Osteogenesis and Barrier Function in the Application of Guided Bone Regeneration. Int. J. Nanomed..

[98] Cárdenas-Aguazaco W., Camacho B., Gómez-Pachón E.Y., Lara-Bertrand A.L., Silva-Cote I. (2023). Electrospun Scaffolds of Polylactic Acid, Collagen, and Amorphous Calcium Phosphate for Bone Repair. Pharmaceutics.

[99] Jeyachandran D., Murshed M., Haglund L., Cerruti M. (2023). A Bioglass-Poly(lactic-co-glycolic Acid) Scaffold@Fibrin Hydrogel Construct to Support Endochondral Bone Formation. Adv. Healthc. Mater..

[100] Rajendran M., Iraivan G., Ghayathri B.L., Mohan P., Chandran K.R., Nagaiah H.P., Selvaraj R.C.A. (2021). Antibiotic Loaded Nano Rod Bone Cement for the Treatment of Osteomyelitis. Recent Pat. Nanotechnol..

[101] Billings C., Anderson D.E. (2022). Role of Implantable Drug Delivery Devices with Dual Platform Capabilities in the Prevention and Treatment of Bacterial Osteomyelitis. Bioengineering.

[102] Zeng M., Xu Z., Song Z.Q., Li J.X., Tang Z.W., Xiao S., Wen J. (2023). Diagnosis and treatment of chronic osteomyelitis based on nanomaterials. World J. Orthop..

[103] Chen Y., Wu X., Li J., Jiang Y., Xu K., Su J. (2022). Bone-Targeted Nanoparticle Drug Delivery System: An Emerging Strategy for Bone-Related Disease. Front. Pharmacol..

[104] Aguilera-Correa J.J., Gisbert-Garzarán M., Mediero A., Fernández-Aceñero M.J., de-Pablo-Velasco D., Lozano D., Esteban J., Vallet-Regí M. (2022). Antibiotic delivery from bone-targeted mesoporous silica nanoparticles for the treatment of osteomyelitis caused by methicillin-resistant Staphylococcus aureus. Acta Biomater..

[105] Andrade S., Ramalho M.J., Santos S.B., Melo L.D.R., Santos R.S., Guimarães N., Azevedo N.F., Loureiro J.A., Pereira M.C. (2023). Fighting Methicillin-Resistant Staphylococcus aureus with Targeted Nanoparticles. Int. J. Mol. Sci..

